# *Staphylococcal* Panton-Valentine Leukocidin Induces Pro-Inflammatory Cytokine Production and Nuclear Factor-Kappa B Activation in Neutrophils

**DOI:** 10.1371/journal.pone.0034970

**Published:** 2012-04-18

**Authors:** Xiaoling Ma, Wenjiao Chang, Cuiping Zhang, Xin Zhou, Fangyou Yu

**Affiliations:** 1 Department of Laboratory Medicine, Anhui Provincial Hospital, Anhui Medical University, Hefei, China; 2 Department of Laboratory Medicine, First Affiliated Hospital of Wenzhou Medical College, Wenzhou, China; Charité-University Medicine Berlin, Germany

## Abstract

Panton-Valentine leukocidin (PVL) is a cytotoxin secreted by *Staphylococcus aureus* and associated with severe necrotizing infections. PVL targets polymorphonuclear leukocytes, especially neutrophils, which are the first line of defense against infections. Although PVL can induce neutrophil death by necrosis or apoptosis, the specific inflammatory responses of neutrophils to this toxin are unclear. In this study, both *in vivo* and *in vitro* studies demonstrated that recombinant PVL has an important cytotoxic role in human neutrophils, leading to apoptosis at low concentrations and necrosis at high concentrations. Recombinant PVL also increased the levels of pro-inflammatory cytokine secretion from neutrophils. The up-regulation of pro-inflammatory cytokines was due to nuclear factor-kappa B (NF-κB) activation induced by PVL. Moreover, blocking NF-κB inhibited the production of inflammatory cytokines. To test the role of neutrophil immune responses during the pathogenesis of PVL-induced acute lung injury, we used immunocompetent or neutropenic rabbits to develop a model of necrotizing pneumonia. Immunocompetent rabbits challenged with PVL demonstrated increased inflammation containing neutrophilic infiltrates. In addition, there were elevated levels of inflammatory cytokines (IL-6, IL-8, TNF-α and IL-10) and NF-κB in the lung homogenate. In contrast, the lung tissues from neutropenic rabbits contained mild or moderate inflammation, and the levels of inflammatory cytokines and NF-κB increased only slightly. Data from the current study support growing evidence that neutrophils play an important role in the pathogenesis of PVL-induced tissue injury and inflammation. PVL can stimulate neutrophils to release pro-inflammatory mediators, thereby causing an acute inflammatory response. The ability of PVL to induce inflammatory cytokine release may be associated with the activation of NF-κB or its pore-forming properties.

## Introduction

*Staphylococcus aureus* infections are increasingly common in the general population and can cause serious infectious diseases, from skin and soft tissue disease to fatal infections [1]. The virulence and relatively facile transmission of *S. aureus* make it a dangerous pathogen and a cause for increased concern. Among several putative determinants of *S. aureus* virulence, Panton-Valentine leukocidin (PVL) is the key toxin and is associated with increased risk of transmission, complications and hospitalization [2]. PVL is a β-barrel pore-forming protein that creates an octamer structure essential for pore formation on host cells [3]–[4]. Both human and rabbit neutrophils are highly sensitive to the pore-forming properties of PVL and rapidly undergo cell death.

Compared with PVL-negative *S. aureus* isolates, PVL-positive isolates are generally more virulent and typically occur in community environments [5]–[6]. *S. aureus* virulence is increasing worldwide and it has become the main cause of severe necrotizing diseases, such as necrotizing pneumonia, severe sepsis and severe skin infections [7]–[9]. Owing to the severe infections caused by PVL-producing *S. aureus*, isolates with high morbidity and mortality rates have arisen despite antibiotic use [10]–[11].

The mechanisms of how PVL may cause tissue injury in necrotizing diseases are unknown. One possibility is that PVL can induce lysis of polymorphonuclear leukocytes resulting in impaired host defense. This would interfere with the clearance of pathogenic organisms and allow further bacterial growth and the increased expression of tissue-damaging exotoxins [2]. However, a recent study indicated that PVL-induced acute lung injury was independent of bacterial survival or replication in the lung [12]. Thus, antibiotic treatments do not significantly reduce mortality. A previous study demonstrated that specific antibodies to LukS-PV (a protein secreted by the PVL gene) induced by intranasal or subcutaneous vaccination could protect animals against acute infection and reduce inflammation [13]. Therefore, PVL may activate target cells to release excessive levels of inflammatory cytokines that may cause acute uncontrolled inflammation and tissue injury [2], [12], [14]. Using microarray profiling and biochemical studies, Zivkovic *et al* [15] showed that PVL can induce highly specific inflammatory transcriptional responses in alveolar macrophages. However, the effect of PVL on the production of inflammatory cytokines in neutrophils is unclear. Therefore, this study investigated the detailed functions and mechanisms of PVL-induced inflammatory cytokine production in neutrophils.

## Materials and Methods

### Ethics statement

Taking of blood samples from humans as well as cell isolation and animal experiments were approved by the local ethics committee (Ethics Committee of the Anhui Provincial Hospital). Human blood samples were taken from healthy volunteers, who provided written informed consent for the collection of samples and subsequent neutrophil isolation. All animals were handled in strict accordance with the recommendations of the Weatherall report, and animal keeping, endotracheal instillation and killing were supervised with the help of the Animal Experiment Center of Anhui Provincial Hospital.

### rPVL production and purification

The pET28a vector (Roche Diagnostics Corp., Switzerland) was used to produce recombinant 6 His-tagged LukS-PV and LukF-PV proteins (the two components of PVL). The sequence was amplified from PVL-positive *S. aureus* isolates using the following primers, close to the coding region of LukF-PV and LukS-PV: *LukF-FW* (5′-acgcGGATCCGCTCAACATATCACACCTGTAAG-3′), *LukF-RV* (5′-accgCTCGAGTTAGCTCATAGGATTTTTTTCCTTAGATTG-3′), *LukS-FW* (5′-acgcGGATCCGAATCTAAAGCTGATAACAATATTGAGAATATTG-3′), and *LukS-RV* (5′-accgCTCGAGTCAATTATGTCCTTTCACTTTAATTTCATGAG-3′). PCR products were digested with XhoI and BamH I (Promega, Madison, WI, USA) and ligated into pET28a. Respective *LukF*-pET28a and *LukS*-pET28a constructs were transformed into *Escherichia coli* DH5α (Invitrogen, Carlsbad, CA, USA), and clones were verified by DNA sequencing. BL21 (DE3) pLys (Invitrogen) were used for expression of pET28a plasmids for 6 h following induction with 0.1 mM isopropyl-β-D-thiogalactoside (Promega). The fusion proteins (6His-LukS-PV and 6His-LukF-PV) were purified by His·Band Purification Kit (Novagen, Darmstadt, Germany) according to the manufacturer's instructions. Finally, proteins were subjected to LPS removal using DetoxiGel columns (Thermo Scientific) until a final LPS concentration of <0.02 EU/ml was ensured.Purity of the fusion proteins were checked by western blot. LukS and LukF were stored at −20°C. Both subunits were mixed at equimolar ratios immediately before addition to cells or rabbits.

### Neutrophil cell isolation

Human neutrophils were isolated from peripheral blood of healthy volunteers with their informed consent and with the ethics committee of the Anhui Provincial Hospital approval. Neutrophils were isolated by dextran sedimentation and centrifugation using Polymorphprep (TBD Co., China), according to the manufacturer's instruction. Erythrocytes were lysed by short treatment of the cell pellet with an ice-cold isotonic NH_4_Cl solution (155 mM NH_4_Cl, 10 mM KHCO_3_, 0.1 mM EDTA, pH 7.4). Cell viability was assessed using the trypan blue exclusion method and a viability of 94% or more was used for experiments. In all cases, cell purity was above 95% as observed following May-Grünwald and Giemsa staining.

### Cell culture and rPVL challenge

Purified neutrophils were cultured in RPMI 1640 culture medium (Gibco-BRL, Gaithersburg, MD, USA) supplemented with 10% heat-inactivated FCS (Gibco-BRL), 100 U/ml penicillin, and 100 µg/ml streptomycin. Twenty-four hours before treatment, cells were seeded into tissue culture plates in triplicate. At the time of treatment, culture medium was replaced with fresh RPMI 1640 containing PBS, 5 nmol/L rPVL or 100 nmol/L rPVL respectively, according to the methods described previously [12], [15]–[16]. For the NF-κB inhibitor assays, isolated neutrophils were further divided into 3 groups designated as group A, B and C. Cells in group A were treated with PBS as a negative control. Cells in group B were directly treated with 100 nmol/L rPVL for 2 h. Neutrophils in group C were pretreated with PDTC (Invitrogen) for 1 h, a potent inhibitor of NF-κB, before the addition of experimental stimuli.

### Observation of cell morphology

After rPVL treatment, the morphology of neutrophils from different groups was observed using a phase contrast microscope (Nikon, Tokyo, Japan). Cells were then collected and examined for morphologic changes by Wright and Giemsa staining using a biological microscope (Nikon). The ultrastructure of PVL- treated neutrophils was examined by transmission electron microscopy.

### ELISA

The amount of cytokines (IL-6, IL-8, IL-10 and TNF-α) released from the culture medium of cells and lung tissue homogenate were determined using specific ELISAs (R&D Systems, Minneapolis, MN, USA) in accordance with the manufacturer's instructions.

### Reverse-transcription (RT) polymerase chain reaction (PCR)

Total RNA extracted from neutrophils were reverse-transcribed for 1 h at 42°C with AMV reverse transcriptase, followed by PCR analysis using the primers as described below (Table 1). During analysis, the amplified transcripts of β-actin were used as an internal control.

**Table 1:** Specification of primers used in RT-PCR.

Target gene	Primer Sequence 5′—3′	Length of production
TNF-α	S: 5′ GTCGGTCACCCTTCTCCA 3′ 18	524 bp
	A: 5′ CAGCCTCTTCTCCTTCCT 3′ 18	524 bp
IL-8	S: 5′ ACATACTCCAAACCTTTCCACCC 3′ 23	175 bp
	A: 5′ CAGCCCTCTTCAAAAACTTCTCC 3′ 23	175 bp
IL-6	S: 5′ GTCCAGTTGCCTTCTCCC 3′ 18	223 bp
	A: 5′ GCCTCTTTGCTGCTTTCA 3′ 18	223 bp
IL-10	S: 5′ ATGCACAGCTCAGCACTGCTCTG -3′ 23	448 bp
	A: 5′ GGAAGAAATCGATGACAGCGCCG -3′ 23	448 bp
β-actin	S: 5′ GGGACCTGACTGACTACCTC 3′ 20	546 bp
	A: 5′ ACTCGTCATACTCCTGCTTG 3′ 20	546 bp

### Western blot

Nuclear proteins (NF-κB P65) were extracted using the Nuclear Protein Extraction Kit (BestBio Corp., Shanghai, China), in accordance with the manufacturer's instructions. The concentration of extracted protein was determined using the BCA method. Western blotting was performed as described previously [12]. Mouse anti-NF-κBp65 antibody was used at a dilution of 1∶500. β-actin antibody served as a control to confirm equal loading. Densitometry index analysis of the bands was achieved using a gel imaging system.

### Development of the necrotizing pneumonia model in rabbits

Rabbits were randomly divided into three groups of five rabbits each. The immunocompetent rabbit treatment group received 25 µg rPVL as previously described [15]. The neutropenia treatment group received two doses of vinblastine (0.75 mg/kg) i.v. at 96 h and 48 h to induce neutropenia, and then 25 µg rPVL was administered into the lungs. Rabbits in the control group received 0.9% saline. All liquids were delivered directly into the lungs of rabbits through a 2.5-mm pediatric endotracheal tube positioned 1 cm above the main stem bronchi. Rabbits were sacrificed at 9 h post-infection.

### Histopathology

Lung tissues were harvested after 9 h post-infection, fixed in 10% formalin, and embedded in paraffin. Paraffin sections of lung tissue were cut to 4 µm thick, and stained using hematoxylin and eosin. Each specimen was analyzed blind by a pathologist.

### Immunohistochemical staining

Immunohistochemical staining was carried out following the manufacturer's instructions. Briefly, following Ag retrieval and blocking, lung sections were incubated with mouse anti-NF-κB FITC (BD Biosciences, San Jose, CA, USA) (1∶100 dilution) at room temperature. After rinsing with PBS for 5 min, the sections were incubated in horse-radish peroxidase-conjugated secondary antibody. Following secondary washing, peroxidase activity was visualized with diaminobenzidine as a chromogen. All sections were rinsed and counterstained lightly with Harris hematoxylin.

### Statistical analysis

Differences between groups were analyzed using unpaired t test or one-way ANOVA. Values are expressed as mean±SD. A value of *p*<0.05 was considered statistically significant.

## Results

### Recombinant PVL is a potent cytolytic toxin for human neutrophils

**Figure 1:**
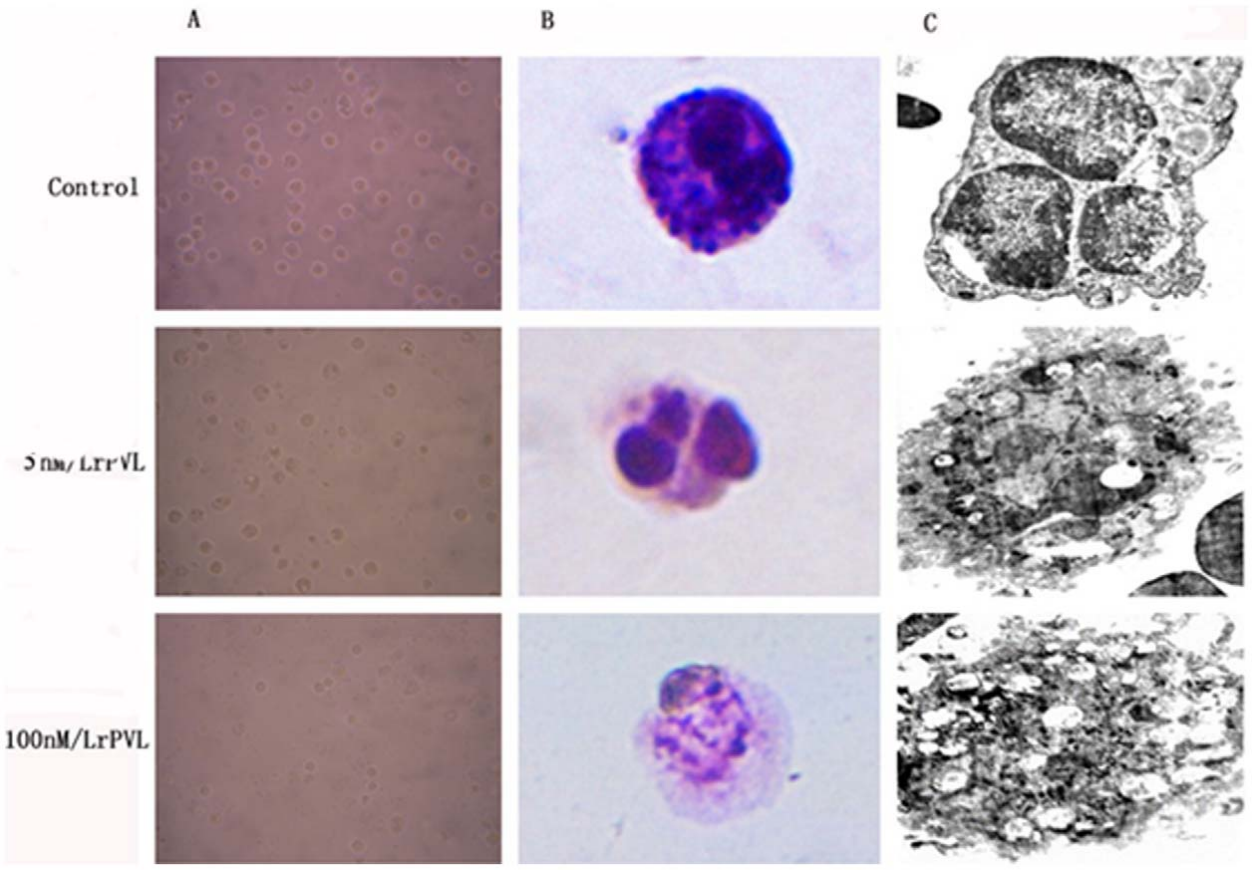
Neutrophil apoptosis and necrosis as a function of the rPVL concentration. Neutrophils were incubated with PBS, 5 nM/L rPVL and 100 nM/L rPVL for 5 h and then observed by (A) phase contrast microscope, (B) biological microscope, or (C) transmission electron microscopy.

The two products of *pvl* expression, rLukS-PV and rLukF-PV, were incubated with neutrophils at equimolar concentrations of 5 nmol/L or 100 nmol/L for 5 h. Compared with the control group receiving PBS, neutrophils showed typical features of apoptosis, with rounding of cells (Figure 1A). In addition, nuclei and pronounced chromatin condensation (Figure 1B) were observed after treatment with 5 nmol/L rPVL for 5 h. In contrast, 5 h of treatment with 100 nmol/L rPVL induced typical necrosis morphology, including swelling, presence of vacuoles and karyorrhexis (Figure 1B).

The morphologic observations were confirmed by ultrastructural examination using transmission electron microscopy. Following treatment with 5 nmol/L rPVL, most neutrophils demonstrated pyknotic nuclei, with a migration of nuclear chromatin, formation of apoptotic bodies, and some vacuoles present in the cytoplasm. In contrast, neutrophils treated with 100 nmol/L rPVL were predominantly necrotic, with loss of cell membrane integrity and many vacuoles present in the cytoplasm (Figure 1C).

### PVL induces neutrophils to release pro-inflammatory cytokines

**Figure 2:**
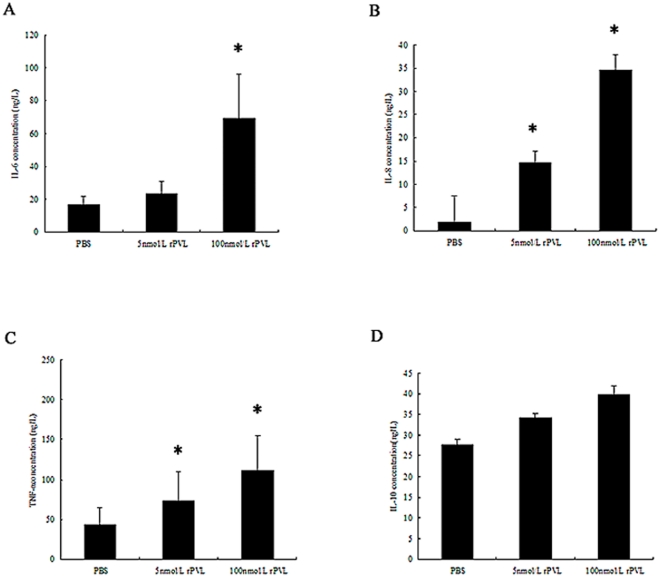
Effect of rPVL on cytokine secretion. 1, control; 2, treated with 5 nmol/L rPVL for 5 h; 3, treated with 100 nmol/L rPVL for 5 h. The supernatants of neutrophils were collected and IL-6 (A), IL-8 (B), TNF-α (C), IL-10 (D) secretion was measured by ELISA. Results represent the mean ± SD of three separate measurements. * *P*<0.05.

As shown in Figure 2, treatment of neutrophils with 5 nmol/L rPVL increased the levels of IL-6, IL-8 and TNF-α in the medium from 16.7 to 23.45 µg/L, 1.8 to 14.7 µg/L, and 43.53 to 72.53 µg/L, respectively. The treatment with 100 nmol/L rPVL resulted in 4.2-fold, 19.3-fold and 2.7-fold increases in IL-6, IL-8 and TNF-α levels respectively, when compared with the control group (Figure 2). However, the effect of rPVL on IL-10 secretion was not significantly different between the control and rPVL treatment groups (Figure 2D).

**Figure 3:**
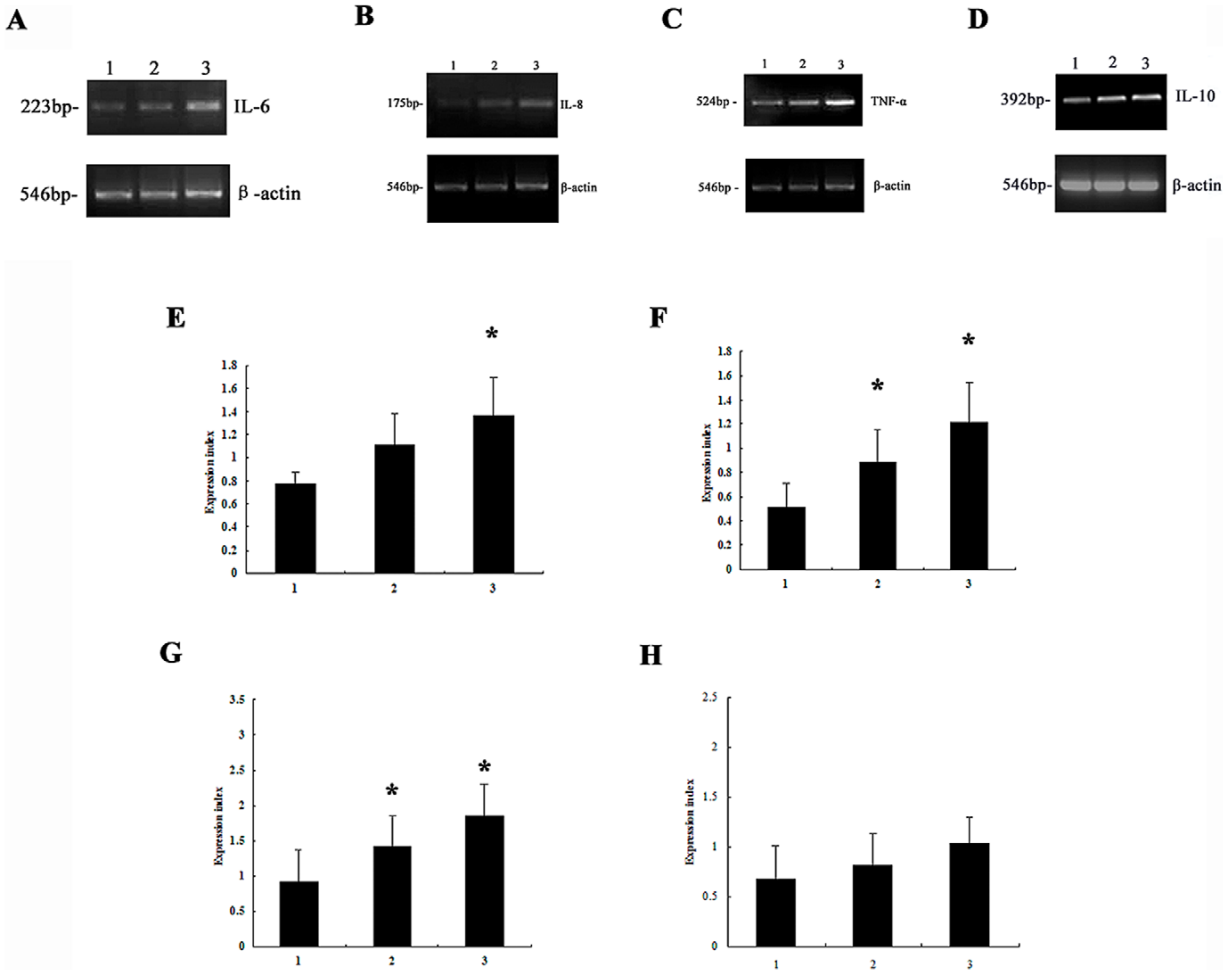
Expression levels of cytokine mRNA in neutrophils. 1, control; 2, treated with 5 nmol/L rPVL for 5 h; 3, treated with 100 nmol/L rPVL for 5 h. Representative agarose-gel photographs showing the expression level of IL-6 (A), IL-8 (B), TNF-α (C) and IL-10 (D) mRNA from neutrophils by RT-PCR analysis. The relative levels of IL-6 (E), IL-8 (F), TNF-α (G) and IL-10 (H) expressed were compared with β-actin levels. * *P*<0.05.

Similar to the protein secretion data, exposure of neutrophils to 5 nmol/L rPVL induced up-regulation of IL-8 and TNF-α mRNA expression. Treatment with 100 nmol/L rPVL increased IL-6, IL-8 and TNF-α mRNA expression by 44.1%, 74.5% and 56%, respectively (Figure 3). In contrast, no significant IL-10 expression was noted following 5 h of infection with rPVL (Figure 3D).

### PVL-induced nuclear factor-kappa B (NF-κB) is important for pro-inflammatory cytokine synthesis

**Figure 4:**
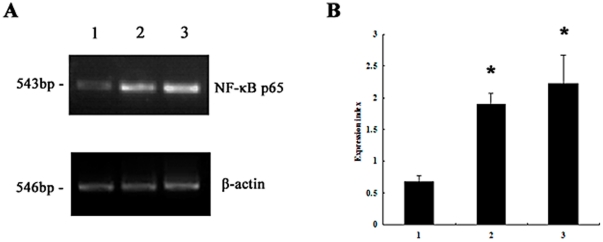
Expression of NF-κB p65 mRNA in neutrophils. Cells were treated with PBS (1), 5 nmol/L rPVL for 2 h (2) or 100 nmol/L rPVL for 2 h (3), respectively. A: Representative agarose-gel photograph showing the expression levels of NF-κB p65 mRNA in polymorphonuclear cells using RT-PCR. B: Relative levels of NF-κB p65 expressed in polymorphonuclear cells. The values indicate the expression indices of the densitometry units relative to the amount of β-actin. Results represent the mean ± SD of three separate measurements. * *P*<0.05.

**Figure 5:**
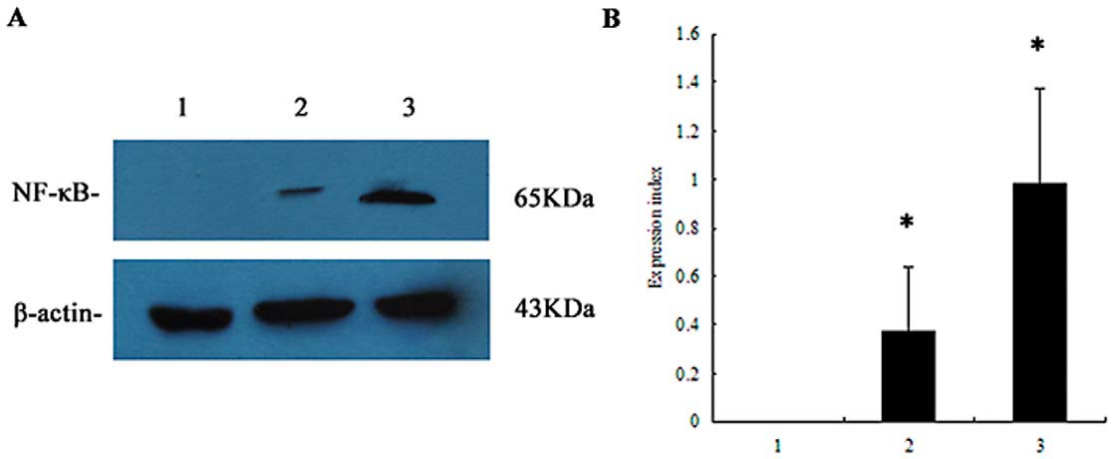
Expression of NF-κB protein in neutrophils. Cells were treated with PBS (1), 5 nmol/L rPVL for 2 h (2) or 100 nmol/L rPVL for 2 h (3) respectively. A: The expression levels of NF-κB proteins in polymorphonuclear cells were analyzed by western blot. B: The relative levels of NF-κB expressed in polymorphonuclear cells. The values indicate the expression indices of the densitometry units relative to the amount of β-actin. Results represent the mean ± SD of three separate measurements. * *P*<0.05.

As shown in Figure 4, following treatment with 100 nmol/L rPVL for 2 h, NF-κBp65 mRNA expression increased 3.3-fold relative to levels in the control group (n = 3, P≤0.05). Western blot analysis showed a higher expression level of NF-κB protein in neutrophils compared with control groups (Figure 5). The mean ± SD expression level of NF-κB protein in the control group, 5 nmol/L rPVL treatment group, and 100 nmol/L rPVL treatment group were 0, 0.28±0.07, and 1.13±0.22, respectively (P≤0.05).

**Figure 6:**
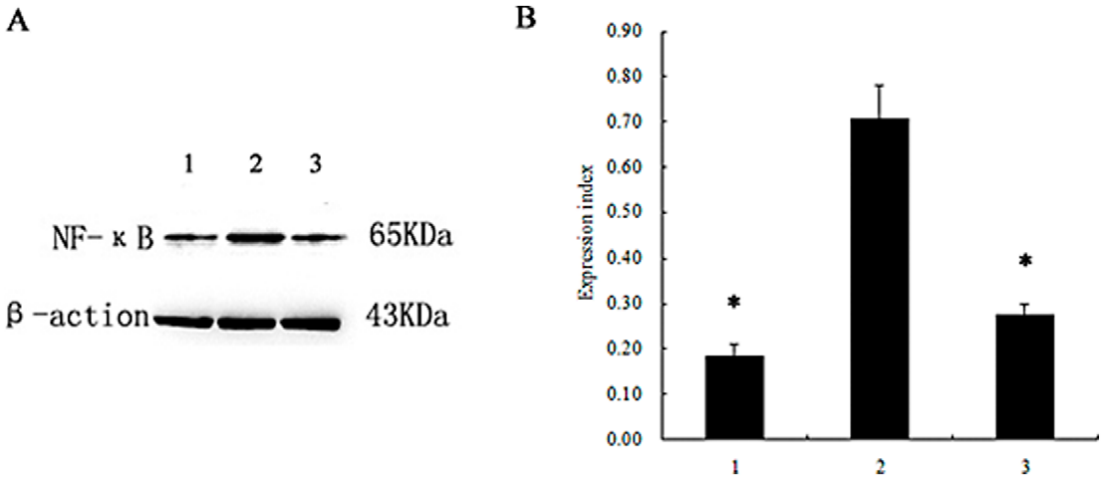
Expression of NF-κB protein in neutrophils after NF-κB inhibition. 1: Cells treated with PBS; 2: cells treated with rPVL; 3: cells incubated with PDTC for 1 h before treatment with rPVL. A: The expression levels of NF-κB proteins in polymorphonuclear cells were analyzed by western blot. B: Relative levels of NF-κB expressed in polymorphonuclear cells. The values indicate the expression indices of the densitometry units relative to the amount of β-actin. Results represent the mean ± SD of three separate measurements. * *P*<0.05.

**Figure 7:**
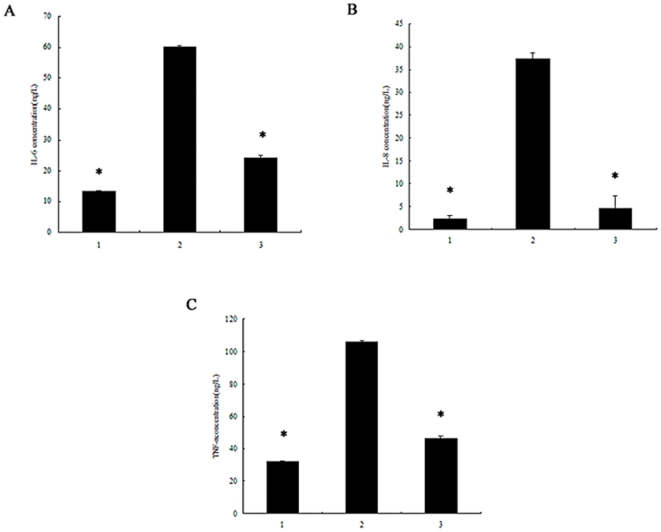
Effect of rPVL on cytokine secretion in neutrophils. 1: Cells were treated with PBS; 2: cells were treated with rPVL; 3: cells pretreated with PDTC for 1 h before treatment with rPVL. The supernatants of polymorphonuclear cells were collected and IL-6 (A), IL-8 (B), TNF-α (C) levels were measured by ELISA. Results shown are representative of three independent experiments. * *P*<0.05.

**Figure 8:**
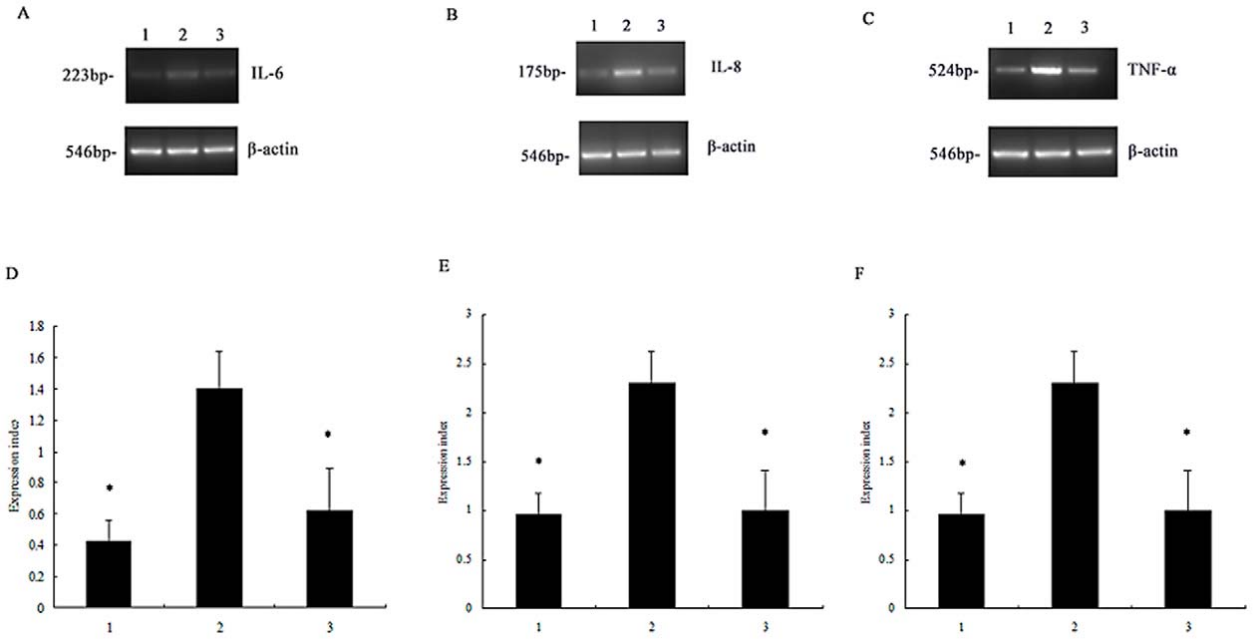
Expression of cytokine mRNA in neutrophils. 1: Cells were treated with PBS; 2: cells were treated with rPVL; 3: cells pretreated with PDTC for 1 h before treated with rPVL. Representative agarose-gel photographs showing the expression levels of IL-6 (A), IL-8 (B) and TNF-α (C) mRNA in polymorphonuclear cells by RT-PCR analysis. The relative levels of IL-6 (D), IL-8 (E) and TNF-α (F) expressed in polymorphonuclear cells are shown. Results represent the mean ± SD of three separate measurements. * *P*<0.05.

To establish whether NF-κB was involved in pro-inflammatory cytokine production, neutrophils were incubated with an NF-κB inhibitor to block its activation before rPVL treatment. Western blot analysis demonstrated that pyrrolidine dithiocarbamate (PDTC) reduced NF-κB activation in neutrophils treated with rPVL (Figure 6). Enzyme-linked immunosorbent assay (ELISA) analysis demonstrated that IL-6, IL-8 and TNF-α production significantly decreased by 59.9%, 73.1%, and 55.9% respectively, in the PVL-treated group compared with the PDTC-pretreated group (Figure 7). RT-PCR analysis showed that pre-treatment with PDTC decreased IL-6, IL-8 and TNF-α mRNA expression induced by rPVL (Figure 8).

### PVL causes inflammatory responses by a neutrophil-dependent mechanism

**Figure 9:**
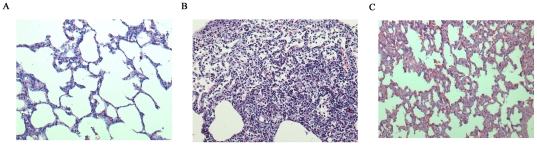
Lung histopathology of rabbits treated with PBS or rPVL. hematoxylin and eosin-stained sections (E–H; magnification 200×) from the lungs of immunocompetent rabbits treated with PBS (A), rPVL (B), or from neutropenic rabbits treated with rPVL (C).

rPVL-treated animals exhibited increased inflammation with neutrophilic infiltrate, necrosis, diffuse alveolar hemorrhage and pulmonary edema. Furthermore, four of five animals displayed areas of necrosis. However, the lung tissues of neutropenic rabbits exhibited mild or moderate inflammation, as only one of five animals had some areas of slight necrosis (Figure 9E).

**Figure 10:**
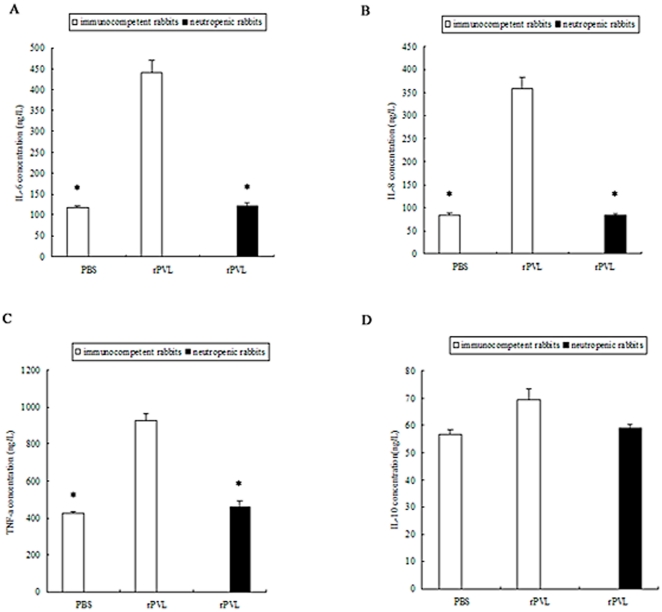
Effect of rPVL on cytokine secretion. IL-6 (A), IL-8 (B), TNF-α (C), and IL-10 (D) from lung tissue homogenate was measured by ELISA. Results shown are representative of two independent experiments. * *P*<0.05.

ELISA analysis demonstrated that IL-6, IL-8 and TNF-α levels increased by 3.7-, 4.3- and 2.2-fold in the lungs of immunocompetent rabbits compared with neutropenic rabbits where there was no increase (Figure 10).

**Figure 11:**
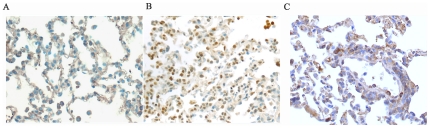
Immunohistochemical staining of NF-κB in lung tissues. NF-κB is mainly expressed in the nucleus of neutrophils. A: Control group negative for NF-κB; B: immunocompetent rabbits inoculated with rPVL; C: neutropenic rabbits inoculated with rPVL.

The expression of NF-κB in the animal models was analyzed by immunohistochemistry. Significant NF-kB nuclear translocation was present in 63.4% (53/80) of neutrophils from immunocompetent rabbits treated with rPVL. NF-κB expression in the lung tissues from neutropenic rabbits treated with rPVL was significantly lower in the nucleus of cells (*P*<0.05) compared with the rPVL-treated immunocompetent rabbits (Figure 11).

## Discussion

A dramatic increase in the number of *S. aureus* infected by bacteriophages carrying the *pvl* gene has triggered an intense search for the underlying factors regarding this [17]–[18]. Previous studies have assayed neutrophil lysis induced by PVL using *S. aureus* culture filtrates *in vitro* [10], [19]–[20]. However, predictions on the relative contribution of PVL to necrotizing pneumonia pathogenesis on that basis are problematic, as PVL concentrations vary considerably depending on the growth media used [11], [21]. Therefore, this study constructed, expressed, and purified recombinant PVL (rPVL). The cytotoxic effect of rPVL was demonstrated using human neutrophils. In our study, rLukS-PV and rLukF-PV were incubated together with neutrophils at equimolar concentrations of 5 nmol/L or 100 nmol/L. Compared with the control group receiving PBS, PMNs viability decreased in vitro, leading to apoptosis at low concentrations and necrosis at high concentrations of rPVL. The morphologic observations were confirmed by ultrastructural examination using transmission electron microscopy.

Taken together, these results indicated that PVL alone was sufficient to induce cell death. This finding confirmed previous studies [2], [4]. PVL can induce neutrophil death *in vitro*; either apoptosis at low concentrations or necrosis at high concentrations. The results also verify that rPVL has the same biological activity as the native PVL protein, providing an experimental basis for the further study of its biological functions.

Evidence from several studies suggests that a complex network of inflammatory cytokines and chemokines play a major role in mediating, amplifying, and perpetuating inflammation [22]–[23]. Regulation of pulmonary inflammation involves an intricate balance of both pro-inflammatory and anti-inflammatory mediators [24]–[25]. Many pro-inflammatory cytokines increase lung injury. Nevertheless, the effect of PVL on cytokine release from neutrophils remains controversial. Previous reports suggest that PVL can induce a continuous increase (over 16 h) of pro-inflammatory IL-8 from human leukocytes [12], [14], [26]. Other studies have reported that PVL significantly increased the production of the anti-inflammatory cytokine IL-10 and slightly decreased the expression of pro-inflammatory cytokines (TNF-α) in neutrophils [27]. In contrast, Christopher *et al* [28] concluded that PVL did not significantly alter the transcription of inflammatory genes in the lung. Therefore, we investigated the inflammatory cytokine release from neutrophils in response to rPVL treatment. In our study, we demonstrated that treatment of neutrophils with rPVL resulted in the gene expression as well as protein production of IL-6, IL-8 and and TNF-α. Whereas, no significant IL-10 expression was noted following treatment with rPVL. These results demonstrate that rPVL can activate neutrophils to release increased levels of pro-inflammatory cytokines, which may cause tissue destruction.

The detailed mechanism of PVL-induced inflammatory cytokine production in neutrophils is still unknown. Previous studies have suggested that PVL induced neutrophil lysis could result in the release of inflammatory cytokines and/or reactive oxygen metabolites [29]–[30]. Using a rabbit model of necrotizing pneumonia, Diep *et al* proposed that PVL could activate polymorphonuclear leukocytes and induce cytokine secretion by an unknown mechanism [12]. Zivkovic *et al* [15] recently demonstrated that PVL could induce the transcription of a small subset of NF-κB-regulated genes and subsequent TNF-α gene expression in primary alveolar macrophages. Whether NF-κB activation is associated with inflammatory cytokine release in neutrophils is still unknown. The current study further investigated the regulatory mechanisms of cytokine production in neutrophils. Following treatment with rPVL, NF-κBp65 mRNA expression increased obviously.Meanwhile, a higher expression level of NF-κB protein in neutrophils compared with control groups showed by Western blot analysis. Neutrophils were further incubated with an NF-κB inhibitor (PDTC) to block its activation before rPVL treatment. IL-6, IL-8 and TNF-α production significantly decreased in the PDTC-pretreated group compared with the PVL-treated group. These data suggested that PVL could induce NF-κB, which is a prerequisite for inflammatory cytokine synthesis.

Taken together, the data suggested that neutrophils might damage lung tissue by secreting inflammatory cytokines, which could be the primary cause of lung injury. To test this hypothesis, rPVL was administered endotracheally into the lungs of immunocompetent or neutropenic rabbits. The severity of histopathology was increased in immunocompetent rabbits compared with the control group (Figure 9D). rPVL-treated animals exhibited increased inflammation with neutrophilic infiltrate, necrosis, diffuse alveolar hemorrhage and pulmonary edema. However, the lung tissues of neutropenic rabbits exhibited mild or moderate inflammation. ELISA analysis also demonstrated that IL-6, IL-8 and TNF-α levels increased in the lungs of immunocompetent rabbits compared with neutropenic rabbits where there was no increase. The expression of NF-κB in the animal models was analyzed by immunohistochemistry. NF-κB expression in the lung tissues from neutropenic rabbits treated with rPVL was significantly lower in the nucleus of cells compared with the rPVL-treated immunocompetent rabbits.

This finding is consistent with a previous report that PVL can induce acute lung injury and lung inflammation, rather than a dependence upon bacterial survival or replication. Furthermore, this suggests that neutrophils can amplify or perpetuate the acute inflammatory response by release of pro-inflammatory cytokines, which can recruit additional neutrophils into the inflamed lung. Taken together, the data indicate that neutrophils are critical for the development of PVL-induced lung inflammation and injury.
